# Modeling and forecasting sex differences in mortality: a sex-ratio approach

**DOI:** 10.1186/s41118-018-0044-8

**Published:** 2018-12-03

**Authors:** Marie-Pier Bergeron-Boucher, Vladimir Canudas-Romo, Marius Pascariu, Rune Lindahl-Jacobsen

**Affiliations:** 1 Center on Population Dynamics, University of Southern Denmark, Odense, Denmark; 2 School of Demography, Australian National University, Canberra, Australia; 3 Department of Epidemiology and Biostatistics, University of Southern Denmark, Institute of Public Health, Odense, Denmark

**Keywords:** Coherent forecasts, Mortality, Female-male differences, Sex ratio, Life expectancy

## Abstract

Female and male life expectancies have converged in most industrialized societies in recent decades. To achieve coherent forecasts between females and males, this convergence needs to be considered when forecasting sex-specific mortality. We introduce a model forecasting a matrix of the age-specific death rates of sex ratio, decomposed into two age profiles and time indices—before and after age 45—using principal component analysis. Our model allows visualization of both age structure and general level over time of sex differences in mortality for these two age groups. Based on a prior forecast for females, we successfully forecast male mortality convergence with female mortality. The usefulness of the developed model is illustrated by its comparison with other coherent and independent models in an out-of-sample forecast evaluation for 18 countries. The results show that the new proposal outperformed the other models for most countries.

## Introduction

Females have had longer life expectancies than males in industrialized societies and females also outlive males in most developing countries today (Austad 2006; Barford et al. 2006; Glei and Horiuchi 2007). This universal disparity has fascinated researchers for decades, and the present consensus considers that the sex gap in life expectancy has biological underpinnings that are modulated by social, behavioral, and environmental conditions (Kingston et al. 2014, 2015; Van Oyen et al. 2013; Oksuzyan et al. 2008; Kalben 2000; Luy 2003).

Biological factors might play a role in sex differences in mortality, but they cannot explain observed variations over time and across countries (Gjonça et al. 1999; Nathanson 1984). These variations have been mainly associated with non-biological factors. It has been established that men engage more in risky behaviors, including a higher level of tobacco, alcohol, and psychoactive substance use, less safe driving, and less healthy nutrition, thus increasing the risks of various morbid conditions and death (Wardle et al. 2004; Waldron 1983). Tobacco consumption is the largest identifiable factor behind the increase in sex differences in mortality in the developed countries, with other risk factors having less significant, separate effects (Lindahl-Jacobsen et al. 2013; Leon 2011; Jacobsen et al. 2008; Katanoda et al. 2008; Preston and Wang 2006; Payne 2004; Pampel 2003; Morris 1955).

Before the 1940s, in industrialized countries, sex differentials in life expectancy were rather constant, but started to increase afterwards due to a faster increase in female life expectancy compared to males (Luy and Wegner-Siegmundt 2013; Thorslund et al. 2013; Morris 1955; Raftery et al. 2014). However, since the 1970s–1980s, the sex gap in mortality has decreased in most industrialized countries. This convergence appeared because females and males had more similar health-related behaviors—e.g., tobacco consumption decreased for males, but increased for females (Lindahl-Jacobsen et al. 2016; Janssen and van Poppel 2015; Trovato and Lalu 2007; Gjonça et al. 2005; Meslé 2004a). One country of exception to this convergence of the sexes is Japan, where the female-male differences in life expectancy continued to increase until the beginning of the 21st century (Meslé 2004a).

**Fig. 1:**
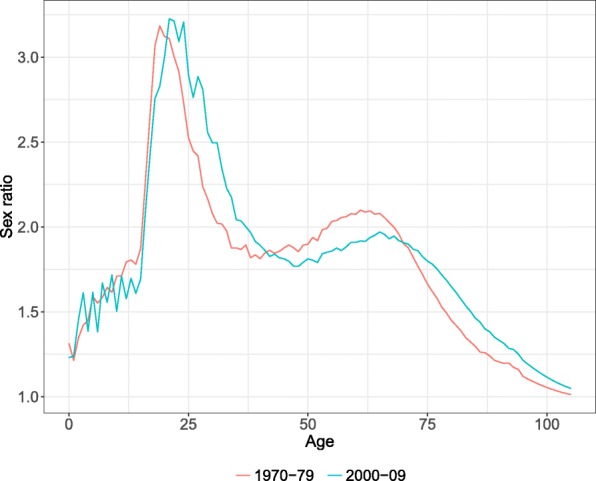
Average sex ratio of the age-specific death rates for 18 countries for the periods 1970–1979 and 2000–2009. *Source: HMD (2017) and own calculations. Note: The selected countries are Australia, Austria, Belgium, Denmark, Finland, France, Germany, Ireland, Japan, the Netherlands, New Zealand, Norway, Portugal, Spain, Sweden, Switzerland, UK, and the USA*

Sex differences in mortality have not, however, declined at all ages for all countries. Meslé (2004a) pointed out that the sex ratio (SR) of the age-specific death rates (ASDR) is generally represented by a peak and a hump. The peak, around age 20, is the result of higher accidental mortality for males. The hump, covering ages from 45 to 75, is the result of higher cancer mortality for males (Meslé 2004a). The SR of the ASDR has been a commonly used indicator to study mortality differences between females and males, as it offers a clearer picture of the disparities by age than the absolute sex differences of the ASDR—i.e., the ratio is less sensitive to mortality level and shows the relative male to female differences (Beltrán-Sánchez et al. 2015; Meslé 2004a; Dublin et al. 1949). Meslé (2004a) noticed that the peak and the hump do not always behave similarly over time. Figure 1 illustrates the peak and the hump of SR at two points in time, showing the average SR for 18 countries for the periods 1970–1979 and 2000–2009. The figure shows that, on average, the peak has increased, while the hump has decreased between 1970–1979 and 2000–2009.

When forecasting mortality by sex, mortality convergence between females and males should be considered. As mentioned by Li and Lee (2005), forecasting separately, the mortality of two populations tends to increase their differences, even when using similar methods. Thus, mortality trends by sex should not be forecasted independently and convergence between sexes should be taken into account. Non-divergent forecasts are often labeled as coherent forecasts. Different models have been introduced to forecast mortality patterns for subpopulations coherently (Li and Lee 2005; Schinzinger et al. 2016; Bohk-Ewald and Rau 2017; Hyndman et al. 2013; Raftery et al. 2012, 2014; Cairns et al. 2011; Torri and Vaupel 2012; Bergeron-Boucher et al. 2017; Pascariu et al. 2017; Janssen et al. 2013; Li 2013; Russolillo et al. 2011; Shang 2016; Shang et al. 2016; Shang and Yang 2017. These models are generally based on the idea of forecasting a mortality trend common to all populations of interest (e.g., an average (Li and Lee 2005), product (Hyndman et al. 2013) or highest level (Torri and Vaupel 2012)) and the population-specific deviation from the common trend. When forecasting mortality for females and males coherently, an extra constraint may also be acknowledged: If females are assumed to have a biological advantage, they can be expected to continue to have lower mortality than males in the future, unless drastic changes occur in terms of health-related behaviors that would disadvantage women or give an advantage to men.

Many coherent forecast models are extensions of the Lee-Carter (Lee and Carter 1992) model (e.g., (Hyndman et al. 2013; Li and Lee 2005; Russolillo et al. 2011; Li 2013)). These models are thus susceptible to carrying some of the Lee-Carter (LC) model limitations, such as its assumption of constant rate of mortality improvement (Booth and Tickle 2008; Lee and Miller 2001). This aspect of the model is due to the use of a fixed age profile of mortality change, which tends to under-predict life expectancy, leading to more biased forecast (Bergeron–Boucher et al. 2017; Booth et al. 2002; Booth and Tickle 2008; Kannisto et al. 1994). Other models, such as those forecasting the life table density of death (Oeppen 2008; Bergeron–Boucher et al. 2017) or using rotation of age profiles (Li et al. 2013; Ševčíková et al. 2016) overcome such a limitation.

In this paper, a new model to forecast male mortality coherently with a female forecast is suggested and builds on the work of Li and Lee (2005), Hyndman et al. (2013), and Shang (2016). Hyndman et al. (2013) forecast the product of female and male ASDR, representing a common trend, and their ratio, representing the difference between sex-specific mortality. The authors state that the product-ratio model is simple and flexible in its dynamic, and the overall accuracy of the model remains comparable to the accuracy of independent models. However, the authors also point out that, with their model, the accuracy of males’ forecast is improved at the expense of that of females (Hyndman et al. 2013). Similar results are found by Shang (2016) when comparing the forecast accuracy between independent functional data model (Hyndman and Ullah 2007) and his coherent multilevel functional data model. In this paper, we suggest using a ratio approach to forecast male mortality, based on a prior female forecast. The accuracy of female independent forecasts will then remain unchanged, and male mortality will be forecast based on their age-specific mortality differences with females. Raftery et al. (2014) and Pascariu et al. (2017) also used a similar strategy, by modeling and forecasting the sex gap in life expectancy. Furthermore, by using a ratio approach based on any prior female forecasts by age, including non-LC type, less biased forecasts for both females and males could potentially be provided. The age-specific sex ratio before and after age 45 are also modeled and forecasted separately, to consider the differences in time trends between the peak and the hump of the SR.

This article is divided into seven sections. In the next section, we introduce the data, followed by the “Methods” section. In the fourth section, the underlying assumptions and interpretation of the parameters of the model are presented. The “Results” section follows, which includes an evaluation of the method, in comparison with other forecasting models, and the mortality forecasts until 2050. The “Discussion and Conclusion” comprise the final sections.

## Data

The data source used is the Human Mortality Database, HMD (2018), which offers high-quality historical mortality data for industrialized countries (Barbieri et al. 2015). The HMD provides data from 39 countries, but the models are tested for low-mortality countries only. Eastern European countries have comparatively high mortality, characterized by breaks and upturns which are more problematic to forecast with common forecasting methods (Meslé 2004b; Fazle Rabbi and Mazzuco 2017). We then selected the remaining countries with data available between 1960 and 2013 and which have a population of more than half a million people. The method is then applied to forecast the mortality of 18 industrialized low-mortality countries: Australia (AUS), Austria (AUT), Belgium (BEL), Denmark (DNK), Finland (FIN), France (FRA), Germany (DEU)^1^, Ireland (IRL), Japan (JPN), The Netherlands (NLD), New Zealand (NZL), Norway (NOR), Portugal (PRT), Spain (ESP), Sweden (SWE), Switzerland (CHE), United Kingdom (UK), and United States America (USA).

We use the HMD period death counts and exposure to risk to calculate the life tables from 1960 to 2013. Mortality above age 95 has been smoothed using a Kannisto model (Thatcher et al. 1998), as used also in the HMD (Wilmoth et al. 2007), to avoid problems with 0 values at higher ages. The multiplicative replacement strategy suggested by Martín-Fernández et al. (2003) to treat zero counts, also applied by Bergeron-Boucher et al. (2017), was used to avoid 0 values at younger ages.

## Methods

We suggest that male mortality be forecasted using the logarithm of the SR of the ASDR. Hyndman et al. (2013) used the SR to forecast mortality, based on a product-ratio method. The authors model and forecast the geometric mean of female and male ASDR (product) and the square root of their ratio using principal component analysis. The product component of their model can be considered as a common trend, similar to that suggested by Li and Lee (2005), and the ratio-component represents the difference between sex-specific mortality. Shang (2016) and Shang et al. (2016) also introduced a similar approach, the multilevel functional data method, which can be seen as an extension of the Li-Lee model and the product-ratio (Hyndman et al. 2013) model, using Bayesian methods (Shang 2016; Shang et al. 2016). These models forecast an average (or product) and the population-specific deviation from the average. More details about these models are provided in Appendix A.

### The sex-ratio (SR) approach

The introduced model builds on the work of the Li and Lee (2005), Hyndman et al. (2013), and the multilevel functional data method (MFDM) of Shang (2016) and Shang et al. (2016). However, the sex ratio model proposed here differs from these models by two main aspects: (1) male mortality is forecasted based on a prior female forecast rather than an average (as also suggested by Raftery et al. (2014); Pascariu et al. (2017)), by modeling and forecasting the sex ratio directly; and (2) the sex ratio before and after age 45 are forecasted separately—i.e., the peak and the hump of the SR, as defined by Meslé (2004a), are modeled separately.

The first modification is applied to avoid losing accuracy in the females’ forecasts (Hyndman et al. 2013; Shang 2016). We do not impose any specific prior female forecast in the model to allow for more flexibility and less bias forecasts. It can be argued that the forecast of the product component in the HBY model and common factor in the MFDM and LL models are similar to the LC model. Thus, these models are susceptible to carry the bias of the LC model. Here, we suggest that female mortality be forecasted with any model forecasting mortality by age, including other models than the LC and its extensions.

The second modification is applied for two reasons. First, sex differences in mortality at young ages can have different trends and causes than those at older ages. We thus model and forecast separate trends for the male excess accident mortality and the male excess cancer mortality (Meslé 2004a). Age 45 is selected as a threshold between the peak and the hump, as the minimum point between the peak and the hump occurs around this age, as discussed in Appendix B. Second, the use of a unique time index for all ages found with a singular value decomposition (SVD) tends to be more strongly influenced by ages having higher values of the centered logged SR (see Eq. (1) below). Appendix B shows that the age group 0–44 tends to have an important impact on a unique time index. As mortality reductions at older ages have more influence on improvements in life expectancy in recent years (Christensen et al. 2009), the use of a unique time index might not capture adequately the changes in the SR at these influential ages.

As a result, a centered matrix of the logged SR of the ASDR by time *t* and age *x* is decomposed into two age profiles and time indices of the males to females ratio:

1a $$ SR_{xt} = ln\left(\frac{m_{xt}^{M}}{m_{xt}^{F}} \right) = \mu_{x} + I(x < 45)\left[\gamma_{t} \phi_{x}\right] + I(x \geq 45)[\!\Gamma_{t} \Phi_{x}] + \epsilon_{xt}  $$

1b $$ m_{xt}^{M} = m_{xt}^{F} e^{SR_{xt}}= m_{xt}^{F} \: e^{\mu_{x} + I(x < 45)\left[\gamma_{t} \phi_{x}\right] + I(x \geq 45)[\Gamma_{t} \Phi_{x}] + \epsilon_{xt}} {,}  $$

where $m_{xt}^{F}$ and $m_{xt}^{M}$ are the ASDR for females and males, respectively, and *ε*_*xt*_ is the error term. The parameter *μ*_*x*_ is the average logged SR and *ϕ*_*x*_ and *Φ*_*x*_ are age profiles of the SR, before and after age 45 respectively. The age profiles indicate the rate of change in the SR, once multiplied by their respective time indices. The parameters *γ*_*t*_ and *Γ*_*t*_ are time indices of the SR and indicate the general level of the sex gap at time *t*. The model parameters are the normalized first singular vectors of the peak and the hump. They are found with a SVD applied to a centered matrix of the logged SR $\left (ln\left (\frac {m_{xt}^{M}}{m_{xt}^{F}} \right) - \mu _{x}\right)$, after being divided into the two selected age groups. The normalization procedure is as suggested by Lee and Carter (1992), so that $\sum \gamma _{t} =1$, $\sum \gamma _{t} =1$, $\sum \phi _{x} =0$, and $\sum \phi _{x} =0$. The term *I* is an indicator function equal to 1 when the associated condition in the bracket is true and 0 when false. An adjustment for the jump-off year has been made using the method of Bergeron-Boucher et al. (2017).

The functional approach of Hyndman and Ullah (2007) used in the HBY (Hyndman et al. 2013) and MFDM (Shang 2016; Shang et al. 2016) models is here set aside, because the second or higher singular vectors (or principal components) are often harder to extrapolate—i.e., we found, in general, that the higher components of the prior models are often not linear and do not increase the explained variance by much (Bergeron–Boucher et al. 2017). Furthermore, in the “Methods” section, we test the SR model assumption (described below) by calculating the correlation between the females and males’ *m*_*xt*_ trends and the in-sample errors. Performing a first analysis on non-smoothed data was thus preferred in order to avoid inflated correlation. However, a functional approach could easily be used, as presented by Hyndman et al. (2013).

### Assumptions

#### Assumption 1: female and male ASDR are correlated and change proportionally

In Eq. (1b), the male ASDR are correlated with the female rates, meaning that, as long as the female ASDR are decreasing, the male ASDR will also keep decreasing. This implies that mortality improvement observed among females will also be noticed among males, but at different levels over ages and time, as determined by the parameters: *μ*_*x*_, *ϕ*_*x*_, *Φ*_*x*_, *γ*_*t*_, and *Γ*_*t*_. The term $ e^{\mu _{x} + I(x \leq 45)[\gamma _{t} \phi _{x}] + I(x > 45)[\Gamma _{t} \Phi _{x}] + \epsilon _{xt}}\phantom {\dot {i}\!}$ should remain higher than 1, ensuring that female mortality is lower than male mortality. To reach coherence, the parameters *γ*_*t*_ and *Γ*_*t*_ should be forecasted as a stationary process. We use ARMA models with the best AIC to forecast *γ*_*t*_ and *Γ*_*t*_, as similarly suggested by Hyndman et al. (2013).

It is important to note that, by using the SR model, we assume not only that female and male ASDR trends are correlated, but that they also decrease proportionally to one another—i.e., there are multiplicative changes. This implies that, even if the model parameters in Eq. (1) stay at a constant value over time, a decrease in female mortality will drive a decrease in male mortality and the absolute sex gap will still be reduced.

#### Assumption 2: independent female forecasts are more accurate than males

To forecast mortality with the model presented in Eq. (1a), the ASDR for one of the sexes should be forecasted beforehand, using any mortality forecasting model by age—for example, the LC model (Lee and Carter 1992). Female life expectancy forecasts are generally more accurate (Booth et al. 2006), and as pointed out by Hyndman et al. (2013), the product-ratio model increases the accuracy for males and decreases it for females. Similar results were also found by Shang 2016. We thus suggest forecasting female mortality beforehand and then forecasting male ASDR, as presented in Eq. (1b). However, in the “Results” section, we also evaluate the performance of the forecast when male mortality is forecasted first and female mortality is forecasted using Eq. (1a).

### Prediction intervals

The prediction intervals (PI) are drawn based on simulations with resampled errors of the model used to forecast the time indices of females and of the SR (*γ*_*t*_ and *Γ*_*t*_). This method allows for a consideration of the two main sources of uncertainty of the model: (1) errors from the SR model presented in Eq. (1b), and (2) the errors from the prior female forecast. More details on how the PI are constructed are given in the “Appendix” C section.

### Comparison with other models

To assess the model’s performance, we compare the SR model, using diverse prior models, with existing forecasting models. We classify the forecast models into three categories: sex-independent models, other sex-coherent models, and the SR coherent model.

1. The sex-independent models are mortality forecasting methods that do not consider the coherence between females and males. We compare five to six models, depending on the sex, in this category:
  - LC: Lee-Carter model (Lee and Carter 1992).
  - LCCC: Li-Lee model (Li and Lee 2005) for country-coherent (CC) forecast, using an average for industrialized countries.
  - FDA: Functional Data approach for mortality forecast (Hyndman and Ullah 2007), using the R package *demography* (Hyndman et al. 2014).
  - CoDa: Compositional Data Analysis model (Oeppen 2008).
  - CoDaCC: CoDa-coherent model for country-coherent forecast, using an average for industrialized countries (Bergeron–Boucher et al. 2017).
  - UN: Bayesian hierarchical model for probabilistic projections used by the United Nations (Raftery et al. 2012; United Nation 2017), using the *bayesLife* R package (Sevcikova et al. 2017). This model is used to forecast female mortality only as performed by the United Nations (Raftery et al. 2014; United Nation 2017).
2. The other sex-coherent (OSC) models are models considering the coherence between sexes, and which have been previously developed. We compare four to five of these models, depending on the sex:
  - LCSC: Li-Lee model for sex-coherent (SC) forecast, i.e., using an average for female and male mortality.
  - CoDaSC: CoDa-coherent model for sex-coherent forecast, also using an average for female and male mortality.
  - HBY: The product-ratio approach of Hyndman et al. (2013), using the R package *demography* (Hyndman et al. 2014).
  - MFDM: Multilevel functional data method (Shang 2016; Shang et al. 2016), using the R package *ftsa* (Hyndman and Shang 2017).
  - UN: Joint probabilistic projections used by the United Nations (Raftery et al. 2014; United Nation 2017), using the *bayesLife* R package (Sevcikova et al. 2017). This model is used to forecast male mortality coherently with the UN-female forecast (Raftery et al. 2014).
3. The SR coherent model is defined in Eq. (1). The prior models used are the five independent models defined in point 1a to 1e. In the following sections, these models have the abbreviation SR followed by the abbreviation of the prior model used. For example, if the male mortality is forecasted with the SR model, with the prior female forecast being the LC model, then this method will be written as SR-LC.

## The model: assumption, interpretation, and goodness of fit

### Female-male mortality correlation

The main assumption behind the model presented in Eq. (1) is that the death rates from both sexes are correlated: when the death rates of females decrease, death rates of males will also decrease. To test if this assumption holds, we calculate the Pearson’s correlation coefficient (R) for the female and male mortality trends over time, at each age. The RV coefficient for females’ and males’ death rate matrices have also been calculated for each country. The RV coefficient is a generalization of the squared Pearson’s correlation coefficient to multivariate data.

**Fig. 2:**
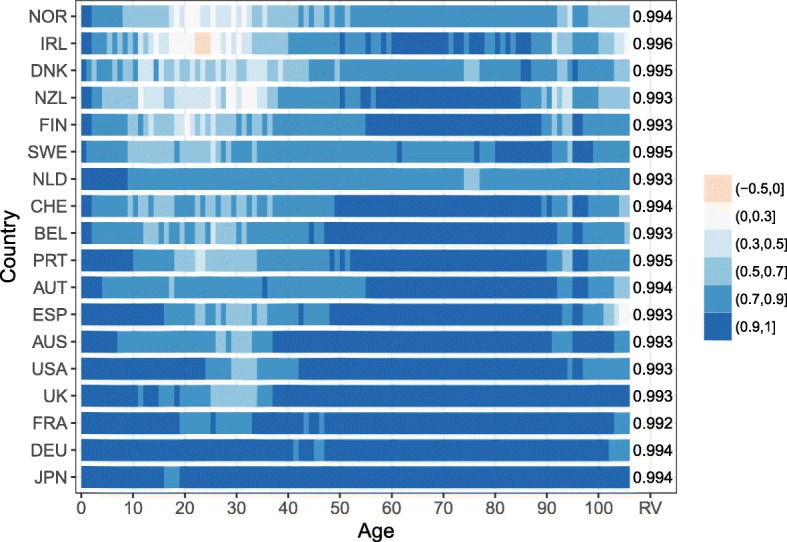
Age-specific correlation coefficient for the female and male death rates trends over time for 18 countries and RV coefficient, 1960–2013. *Note: The countries are ordered from low to high averaged correlation coefficient over age*

For all countries and at almost all ages, the R is positive, meaning that female and male mortality trends are going in the same direction. Figure 2 shows that the female-male trends are strongly correlated (*R*>0.7) between ages 0 and 10, and between ages 40 and 90 for most countries. Only Denmark and the Netherlands show a weaker correlation between ages 70 and 80, but it can still be considered a moderate correlation (0.5<*R*<0.7). The RV coefficient for each country also suggests a strong correlation between females’ and males’ mortality matrices, with a value above 0.99 for all countries.

Between ages 10 and 40, the R is considered strong for five countries (Austria, France, Germany, Japan, and the Netherlands) and shows a strong to moderate correlation for eight other countries. However, the remaining five countries, i.e., Denmark, Finland, Ireland, New Zealand, and Norway, recorded a relatively weak correlation between female and male mortality trends at these ages (− 0.1<*R*<0.5). Only Ireland between ages 24 and 26 had a negative R. Two explanations can contribute to understanding the weak female-male correlation at these ages for these five countries: (1) their populations are relatively small and more variation is recorded at these ages where mortality is low and (2) stagnation, slower decrease, and even an increase of the mortality trends for one of the sexes are observed, while the mortality trends of the other sex have been decreasing. These results might weaken the underlying assumption of the model. However, the number of deaths between ages 10 and 40 is often small—for example, less than 4.5% of the deaths occurred between these ages in 1960, and less than 2.5% in 2013, for Denmark, Finland, Ireland, New Zealand, and Norway. The errors in modelling and forecasting mortality at these ages should have a lesser impact on life expectancy changes. Thus, it is reasonable to assume that female and male mortality trends are correlated.

### Interpretation of parameters

The parameter *μ*_*x*_ is the age-specific average logged SR. It captures the average shape and level of the logged SR for each country. The time indices and age profiles indicate how *μ*_*x*_ is altered at each age over time. The interpretation of the time indices (*γ*_*t*_ and *Γ*_*t*_) and the age profiles (*ϕ*_*x*_ and *Φ*_*x*_) in Eq. (1) are connected. The age profiles indicate the rates of change of the age-specific SR, once multiplied by the time indices. The time indices are indices of the general level of the SR over time. Once combined, the age profiles and time indices tell us the direction and intensity of the SR change over time, at each age. The interpretation of each combination of parameters are as follows:

- If *ϕ*_*x*_ and *Φ*_*x*_ are positive, and *γ*_*t*_ and *Γ*_*t*_ are increasing, the age-specific SR is increasing.
- If *ϕ*_*x*_ and *Φ*_*x*_ are positive, and *γ*_*t*_ and *Γ*_*t*_ are decreasing, the age-specific SR is decreasing.
- If *ϕ*_*x*_ and *Φ*_*x*_ are negative, and *γ*_*t*_ and *Γ*_*t*_ are increasing, the age-specific SR is decreasing.
- If *ϕ*_*x*_ and *Φ*_*x*_ are negative, and *γ*_*t*_ and *Γ*_*t*_ are decreasing, the age-specific SR is increasing.

**Fig. 3:**
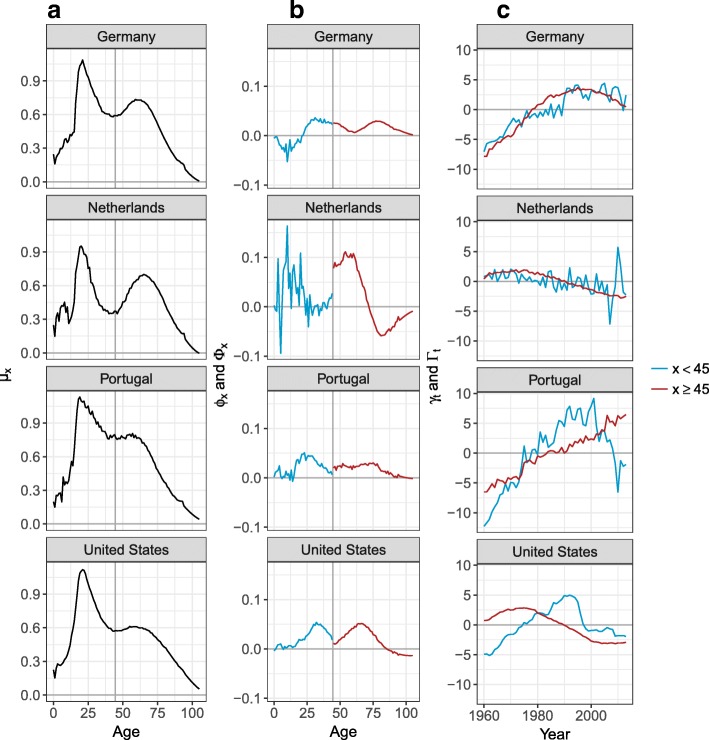
Model parameters— *ϕ*_*x*_ and *γ*_*t*_ in blue and *Φ*_*x*_ and *Γ*_*t*_ in red—for Germany, the Netherlands, Portugal, and the United States. **a** Average logged SR, **b** Age profiles, **c** Time indexes

The age profiles and time indices differ between countries. Figure 3 shows the parameters for Germany, the Netherlands, Portugal, and the USA, as they represent well the different possible patterns observed. If we first look at the Netherlands, the average logged SR shows a clear peak and a clear hump. The peak has been decreasing (decreasing *γ*_*t*_ and positive *ϕ*_*x*_) over all the years selected and the decrease has been more pronounced before age 25. Between age 25 and 44, the SR stayed approximately constant, as *ϕ*_*x*_ is close to 0. The SR have been decreasing between age 45 and 70 since the 1970s. However, they have been increasing after age 70, represented by a negative *Φ*_*x*_ and decreasing *Γ*_*t*_. Such patterns of *Φ*_*x*_, i.e., positive and then negative, generally represent a shift of the hump towards older ages.

When looking at Portugal and the USA, *μ*_*x*_ has a less pronounced hump. For both these countries, the SR between age 0 and 44 have been increasing until the mid-1990s, and since started to decrease. However, the SR after age 45 have been behaving differently between these two countries. The SR for Portugal at these ages have been increasing over the observed period. At these same ages, the SR for the USA have been decreasing since the late 1970s and have leveled off since 2000.

Finally, when looking at Germany, *μ*_*x*_ is also represented by a clear peak and a clear hump. Between age 0 and 25, the SR have been decreasing, but have been increasing between age 25 and 45. The SR above age 45 have been increasing until the late 1980s and since started to decrease.

As mentioned previously, we estimated an age profile and time index for the peak and the hump of the SR. This strategy is used because the time indices sometimes behave differently. As shown in Fig. 3, *γ*_*t*_ and *Γ*_*t*_ for Portugal and the USA have different trends, stressing the need to use separate parameters for these age groups, as further shown in the Appendix B section.

### Goodness of fit

**Fig. 4:**
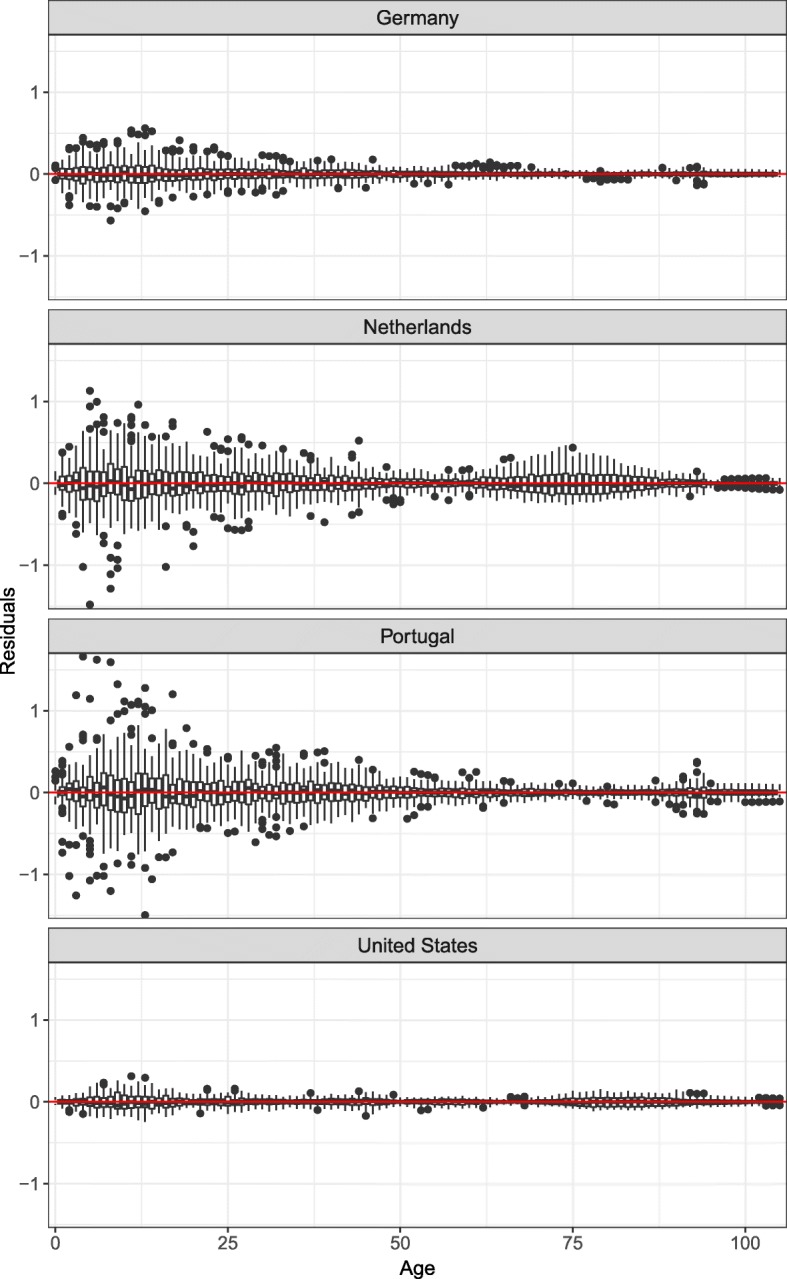
Box plots of the model residuals for Germany, the Netherlands, Portugal, and the United States

To assess the goodness of fit of a model, the box plot of residuals has been considered a useful tool, more than the explained variance (Russolillo et al. 2011; Renshaw and Haberman 2003). Figure 4 plots the residuals of the SR model by age. The box plots show that the residuals have symmetric patterns at most ages, with the medians centered around 0, suggesting that the model generally estimates quite well the SR trends at each age. The figure also shows that the residuals are more important at younger than at older ages. However, for the Netherlands and the USA, the residuals between ages 65 and 90 are more important than at some earlier ages.

**Fig. 5:**
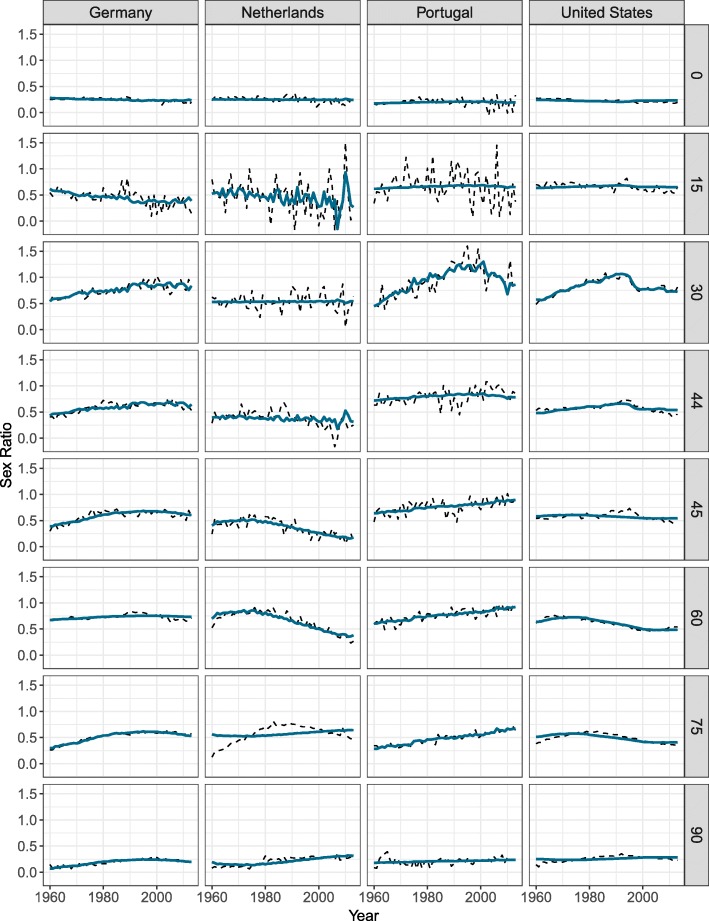
Sex ratio observed (dashed) and fitted (full line) with the SR model for Germany, the Netherlands, Portugal, and the United States at ages 0, 15, 30, 44, 45, 60, 75, and 90

Figure 5 helps in understanding these patterns. The figure shows the SR trends observed and fitted with Eq. (1) at specific ages. More random variation is observed among the SR at young ages, explaining the greater residuals. While the model suggested in Eq. (1) fits quite well with the data for Germany and Portugal at most ages, the residuals are more important for the Netherlands, especially between ages 60 and 90. As mentioned earlier, *Γ*_*t*_ for the Netherlands started decreasing in the 1970s. However, this turning point in the SR trends is not the same at all ages. More precisely, the turning point occurred later in time for older ages. This generally produced a shift in the hump of *μ*_*x*_. As mentioned earlier, this pattern will be reflected by a positive *Φ*_*x*_ at younger ages and a negative *Φ*_*x*_ at older ages, when *Γ*_*t*_ is decreasing. As shown in Fig. 5, the introduced model presents more challenges in modeling such patterns. Similar phenomena were observed for Norway and moderately so for the USA, Australia, Great Britain, and New Zealand.

## Results

### Out-of-sample evaluation

**Fig. 6:**
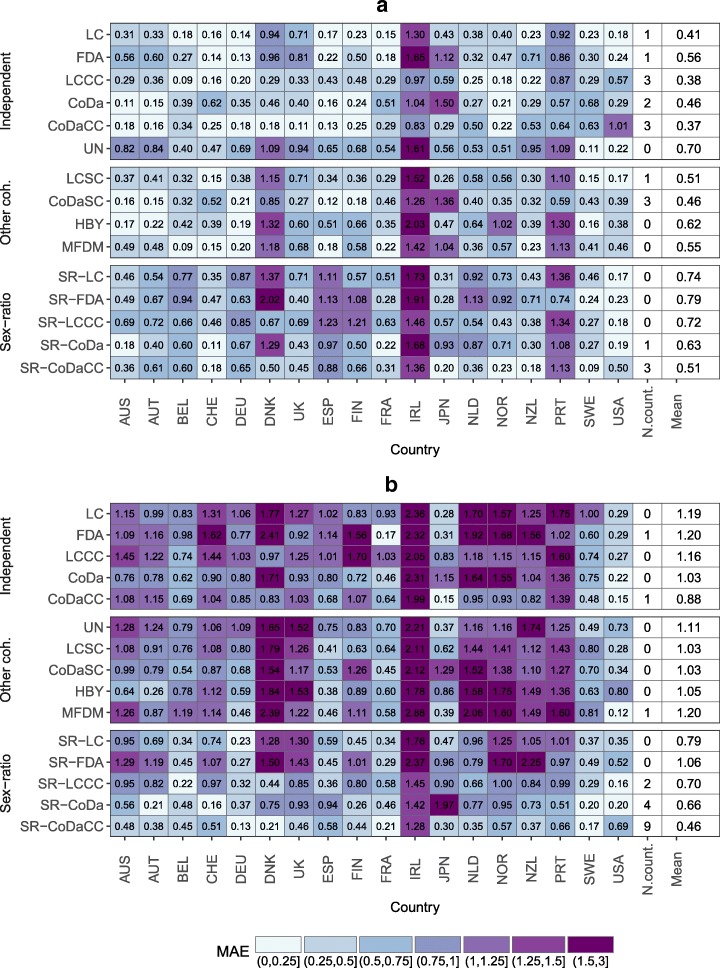
Mean absolute error (MAE) on forecasting the life expectancy at birth using different models (and prior models of the opposite sex for the sex ratio) for the period 1999–2013, mean over countries by model and number of countries with the lowest MAE by model, 18 industrialized countries. **a** Females and **b** Males

**Fig. 7:**
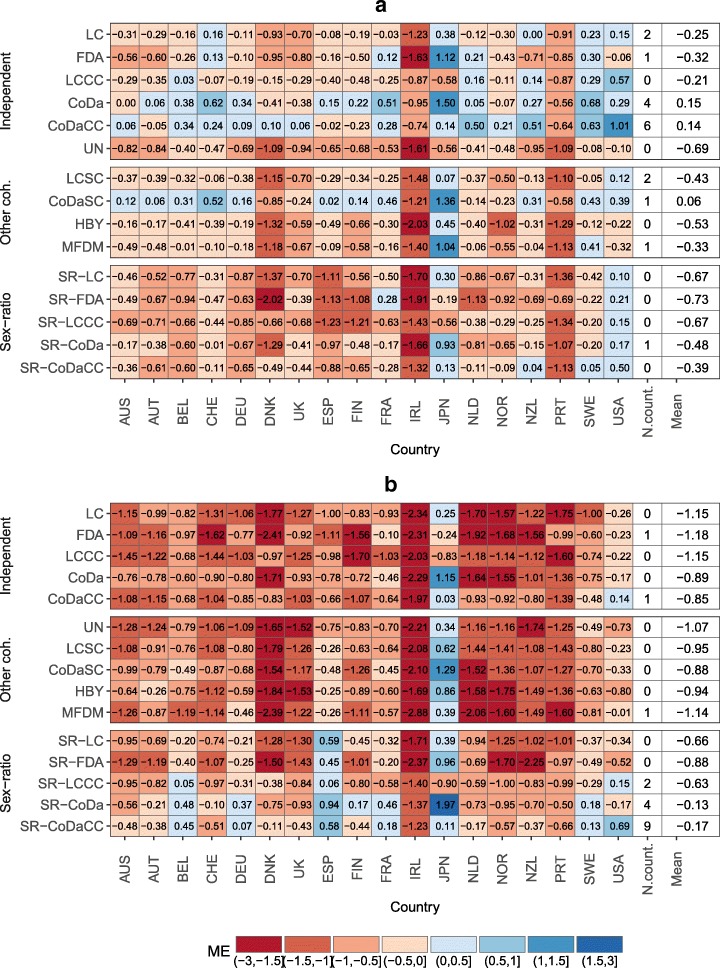
Mean error (ME) on forecasting the life expectancy at birth using different models (and prior models for of the opposite sex the sex ratio) for the period 1999–2013, mean over countries by model and number of countries with the lowest ME by model, 18 industrialized countries. **a** Females and **b** Males

To evaluate the performance of the proposed model, in comparison with the independent and other coherent models listed in the “Comparison with other models” section, we forecast the life expectancy over a 15-year horizon, i.e., from 1999 to 2013, based on the reference period 1960–1998, with all models. Figure 6 presents the mean absolute error (MAE) and Fig. 7 presents the mean error (ME) for the forecast life expectancy. The former is a measure of accuracy, while the latter is a measure of bias of the forecast.

Figure 6a shows that the independent models would have been, on average, more accurate in forecasting female life expectancy between 1999 and 2013, especially the LCCC and CoDaCC models. The other sex-coherent models and the sex ratio model tend to offer somewhat poorer accuracy. However, independent models would have outperformed the sex-coherent models for only 56% of the countries (10 out of 18 countries) for females. Figure 7a shows that the other coherent models and the sex ratio models tend to increase the bias, which is already present in some of the independent models. The LC and LCCC are known to produce too pessimistic forecasts of life expectancy, as shown by a negative ME (Booth and Tickle 2008; Booth et al. 2002; Bergeron–Boucher et al. 2017; Kannisto et al. 1994). Using a sex-coherent model based on an average—e.g., LLSC, CoDaSC, MFDM, and HBY—tends to pull the female forecasts towards the male and to underestimate even more their life expectancy at birth, when compared with the independent models. The CoDaSC models, however, benefit from this “pulling effect” towards the average as the CoDa model tend to overestimate life expectancy over the selected period for females. Independent models would have produced least bias forecast for 72% of the countries (13 out of 18 countries).

The results for males differ from those for females. The independent models perform rather poorly, under-predicting life expectancy. The coherent models tend to perform better, and especially the SR model. Using an SR model would have offered the most accurate forecasts for males for 15 out of 18 (83%) countries, with the exceptions being France (FDA), Japan (CoDaCC), and the USA (MFDM). Regardless of the prior female forecast model, the SR model would have generally increased the accuracy and reduce the bias of the male forecasts for the period 1999–2013. The advantage of the SR model is especially visible when the model is compared with an independent or other sex-coherent counterpart, e.g., when comparing the SR-LC models with the LC and LCSC models, or the SR-CoDa with the CoDa and CoDaSC. However, the SR model still tends to under-predict life expectancy for males, on average, but the bias is greatly reduced compared with the other sex-coherent and independent models.

**Fig. 8:**
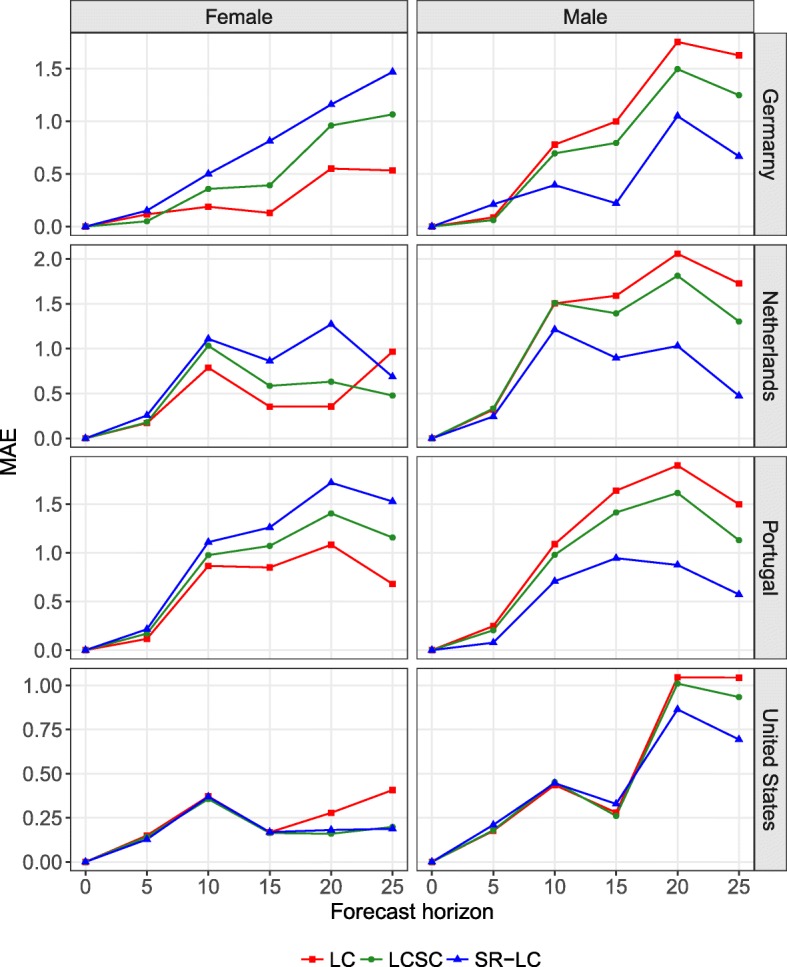
Mean absolute error (MAE) on forecasting the life expectancy at birth for a forecast horizon of 5, 10, 15, 20, and 25 years with the last year of the forecast period being 2013 with the LC, LCSC, and SR-LC models, for Germany, the Netherlands, Portugal, and the United States, females and males

Figure 8 shows an example of MAE for different forecast horizons, with the last year of the forecast period being 2013 for the LC, LCSC, and SR-LC models. For example, if the forecast horizon is 10, the forecast period is 2004–2013 and the reference period is 1960–2003. The figure confirms the results of Fig. 6 for different forecast horizons. Independent models tend to produce more accurate forecasts for females, except for the USA and the Netherlands with a forecast horizon of 25 years. As mentioned earlier, coherent models based on an average (or product) trends—e.g., LLSC, CoDaSC, MFDM, and HBY—tend to decrease accuracy for females, but to increase it for males. For males, the SR model would have been the most accurate for most forecast horizons for the four selected countries. Similar results are shown in Fig. 13 of the Appendix D section, when comparing the CoDa, CoDaSC, and SR-CoDa models.

Results from Figs. 6, 7 and 8 suggest that forecasting female mortality using independent models and then using the SR model presented in Eq. (1) to forecast male mortality coherently with the selected prior female forecast would have been the optimal solution among the models compared.

### Mortality forecasts until 2050

According to the results in Figs. 6 and 7, the CoDaCC model would have been the most accurate and least biased but one (after CoDaCS) model to forecast females’ mortality. Furthermore, using this same model as prior female forecasts when forecasting male mortality with the SR model would have been the most accurate and second least biased strategy for males’ forecasts. In this section, we will use the CoDaCC model to forecast female mortality until 2050. For the male forecasts, we thus use the SR-CoDaCC (Eq. (1)).

**Fig. 9:**
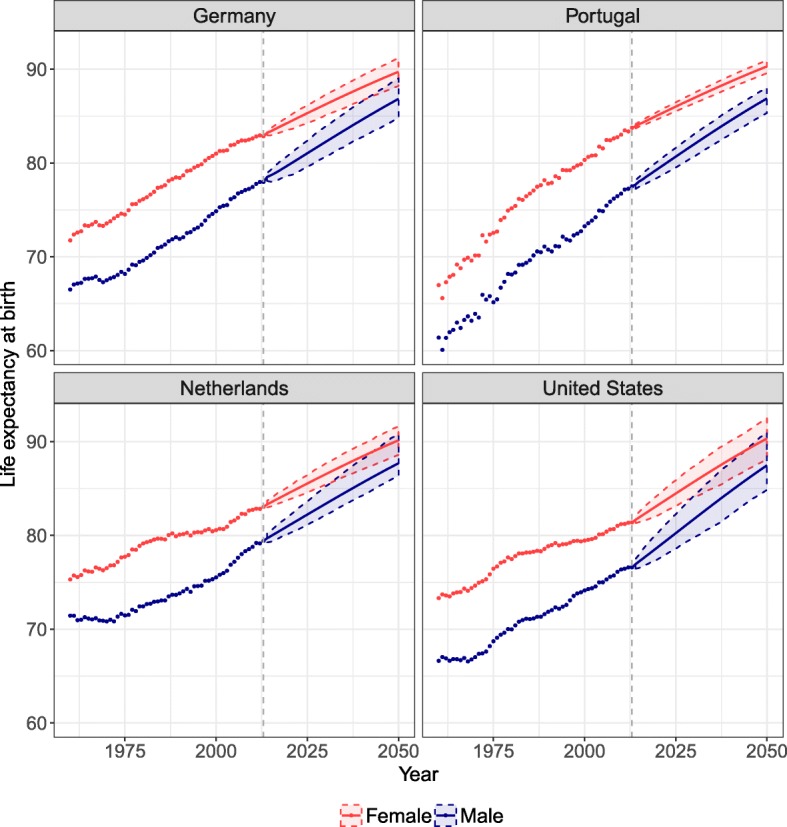
Life expectancy at birth observed (dots) and forecast (lines) from 2013 to 2050 with the CoDaCC model for females and the SR-CoDaCC model for males with their 80% prediction intervals, for Germany, the Netherlands, Portugal, and the United States

Figure 9 shows the life expectancy at birth observed and forecast for Germany, the Netherlands, Portugal, and the USA. The reference period is 1960–2013, and the mortality is forecast until 2050. The SR model allows male life expectancy at birth to catch up with female life expectancy. As *γ*_*t*_ and *Γ*_*t*_ are forecast to eventually reach a constant, male mortality stays higher than female mortality in the forecast.

By using a forecast model for females that considers coherence between countries, this coherence is also reflected in the male forecast when using the SR model, as shown in the Appendix E section. In 2013, the range of life expectancy at birth across countries for males was 76.6–80.6, with a difference between the maximum and minimum values of 4.0 years. By 2050, we predict that the range will be 3.3 years, with a maximum life expectancy of 90.1 for Japan and a minimum of 86.8 for Germany. The SR model thus has the ability to preserve in the male forecasts the coherence among countries integrated in the female forecasts. Similar results are also found if the LCCC model is used as the prior female forecast.

**Fig. 10:**
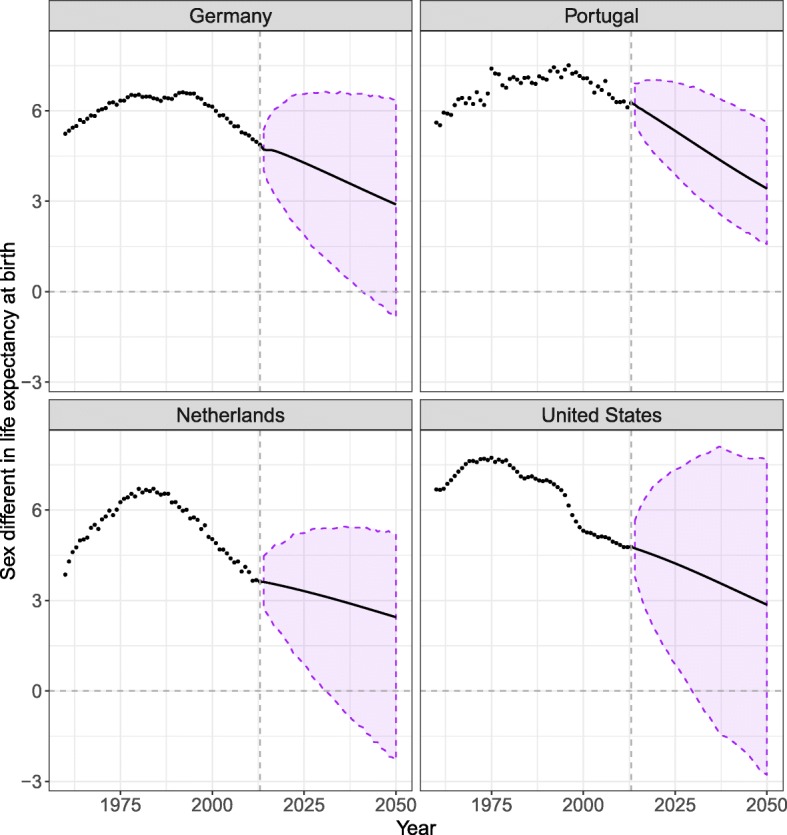
Sex differences in life expectancy at birth observed (dots) and forecast (lines) from 2013 to 2050 resulting from forecasting females’ mortality with the CoDaCC model and that of males with the SR-CoDaCC model, with the 80% prediction intervals, for Germany, the Netherlands, Portugal, and the United States

Figure 10 shows the sex differences in life expectancy at birth observed and forecast for the four selected countries. The forecasts predict that females’ and males’ life expectancy will keep converging over the forecast period. By 2050, the models predict that the sex differences in life expectancy should be between 2.2 (New Zealand) and 3.5 (Japan) years for all 18 countries. We also tested the model for longer forecast periods and found that sex differences in life expectancy will converge towards 0, without crossing this limit. The model thus preserves the female mortality advantage.

Figure 9 shows that the PI for males are wider than for females, due to the fact that the forecast for males, when using Eq. (1), includes more sources of uncertainty, as detailed in the Appendix C section. Furthermore, we see in Fig. 9 that the PI of females and males sometimes cross, as further shown by a negative PI after a certain year in Fig. 10. Even if the SR model ensures that females keep their advantage in the forecasts, no such constraints are included in the PI calculation so that the lower PI bound for females stays higher than the upper PI bound for males. Such constraints could potentially be added. However, it could be possible for males to have lower mortality than females; for example, if women’s tobacco consumption were to increase and exceed that of men.

## Discussion

In this article, we introduced a new model to forecast male mortality coherently with a prior female forecast by age. In an out-of-sample forecast, our model would have been able to predict more accurately the recent male mortality trends than other sex-coherent or sex-independent models, while preserving the female advantage in the forecasts.

The model hypothesizes that male mortality evolves proportionally to female age-specific death rates. This assumption implies that females and males benefit from similar improvements in living conditions and health care, but also suffer similar obstacles to bring mortality rates further down. However, due to different biological and non-biological factors, male mortality stays at higher levels. These sex differences in mortality are determined by the model parameters. As the SR model assumes a proportional decrease of the ASDR of females and males, the absolute difference between females and males will continue to decrease, as long as the females’ ASDR decreases. Under this assumption, the limit to the sex difference in life expectancy is 0. In order to have a limit higher than 0 with the SR model, assumptions have to be made about the lower level that the death rates at each age can reach.

By forecasting females first, independently from males, the model also implies that the common mortality improvements between sexes are best perceived and estimated by the female mortality trends. Raftery et al. (2014) and Pascariu et al. (2017) also used a similar strategy to forecast the life expectancy gap between female and male. Our results confirm that commonly used forecasting models forecast the female mortality trends more accurately than those of males. As mentioned previously, the LC model and its extensions often carry a negative bias and thus tend to underestimate future life expectancy. This bias is especially visible for males. The CoDa model and its coherent extensions are less biased, but still tend the underestimate future life expectancy for males. These results can raise questions about how adequately these models can capture mortality trends and extrapolate them. The SR model can thus be seen as a flexible method to reduce the bias for males, without losing accuracy in the females’ forecast.

By using a prior female forecast instead of an average, the accuracy of the male forecast depends on the accuracy of the selected forecast model for females. As a consequence, the uncertainty of the female forecast should be reflected in the male forecast, leading to wider PI for males than for females. Despite this limitation, the SR model has shown to increase greatly the accuracy of male forecasts. Its flexibility in terms of prior model can be an advantage, allowing the use of a model that is less biased than the LC. Furthermore, the coherence between countries imposed by using a female forecast model considering coherence among these populations is reflected in the male forecasts, when using the SR model. The SR model can thus allow for both sex and country-coherent forecasts.

A limitation of the model is the absence of covariates to estimate the age-specific SR changes over time. Sex differences in mortality are determined by the differential risk factors between females and males associated with health-related behaviors (Kingston et al. 2014, 2015; Van Oyen et al. 2013; Oksuzyan et al. 2008; Trovato and Lalu 2007; Gjonça et al. 2005; Meslé 2004a; Kalben 2000). For example, a reasonable statement would be that forecasting sex differences in mortality should be based on disparities in tobacco and alcohol consumption between females and males (Janssen et al. 2013). These patterns are, however, often harder to forecast than the aggregated measures; their relationship with mortality is often miscalculated and assumptions about future behaviors are often required (Raftery et al. 2014; Booth and Tickle 2008). Until reasonable strategies to overcome these limitations are found, forecasting aggregated measures tends to provide more reliable forecasts (Alho 1991; Wilmoth 1995). Also, the model cannot capture selection effects acting on specific cohorts and how they affect time trends in mortality and sex ratios. However, such effects will tend to be population-specific and not within the scope of the presented SR model, which aims to introduce a general forecast approach based on sex differences in mortality for low mortality countries.

Given that our model does not include the actual risk factors responsible for sex differences in mortality, the model parameters could be seen as proxy of the effect of the combined risk factors on sex differences in mortality. Once the age profiles are combined with their respective time indices, we can approximate how these age-specific effects are changing over time. By using two time indices, we differentiate between the changes in the SR before and after age 45. Age 45 was used as the threshold because it separates the peak and the hump of *μ*_*x*_, and the accidental excess mortality from the cancer excess mortality for males (Meslé 2004a). As shown in the “Interpretation of parameters” section, time trends for these two age groups sometimes have different patterns. More age groups could be used if judged necessary, e.g., to differentiate the SR pattern for infancy from the other age groups.

We make the hypothesis that, due to their biological advantage, females should maintain lower mortality than males in the future. Additionally, despite the fact that females’ and males’ health-related behaviors have become more similar in recent years, males are still more disadvantaged by these non-biological factors, under current observations (Trovato and Lalu 2007; Meslé 2004a; Wardle et al. 2004). However, under certain conditions, males could have lower mortality than females, for example, if females increase in tobacco consumption were to exceed that of males while all the other risk factors associated with sex differences in mortality remain constant. Our model could be adapted to such a scenario, if believed reasonable, by forecasting the time indices as non-stationary processes and so that, in Eq. (1b), the expression $\phantom {\dot {i}\!}e^{\mu _{x} + I(x \leq 45)[\gamma _{t} \phi _{x}] + I(x > 45)[\Gamma _{t} \Phi _{x}] + \epsilon _{xt}}$ stands between 0 and 1.

## Conclusion

A new model to forecast male mortality coherently with a female forecast is introduced. The SR model has proved to be a flexible model, by allowing the use of many models to forecast female mortality by age as prior and to forecast male mortality coherently with it, including less biased models than the Lee-Carter model and country-coherent models. It also allows for a differentiation between the SR trends due to accidental and cancer male excess mortality. The model acknowledges the female mortality advantage at all ages among industrialized countries and preserves this in the forecast. It is shown that the SR approach to forecasting mortality would have increased the accuracy of the male forecast for the period 1999–2013 for 83% of the selected countries.

## Appendix A: Other models

In this section, a brief summary of some of the models mentioned in the paper is presented.

### Lee-Carter model (LC)

2 $$ ln\left(m_{xt}\right) = \alpha_{x} + \beta_{x} \kappa_{t} + \epsilon_{xt}  $$

with

- *m*_*xt*_ is the death rates at age *x* and time *t*.
- *α*_*x*_ is the age-specific average of the logged death rates.
- *β*_*x*_ is the normalized first singular vector of the age mode found with an SVD applied to the center log (*m*_*xt*_) matrix.
- *κ*_*t*_ is the normalized first singular vector of the time mode found with an SVD applied to the center log (*m*_*xt*_) matrix.
- *ε*_*xt*_ is the error term.

### Functional data approach (FDA)

The functional data approach (Hyndman and Ullah 2007) expand on the Lee-Carter model:

3 $$ f_{xt} = \mu_{x} + \sum\limits_{k=1}^{K}\beta_{tk} \phi_{xk} + \epsilon_{xt}  $$

with

- *f*_*xt*_ is the smoothed logged death rates at age *x* and time *t*, using weighted penalized regression splines.
- *μ*_*x*_ is the age-specific average of the logged death rates.
- *ϕ*_*xk*_ is a set of orthonormal basis functions found with a robust functional principal component analysis.
- *β*_*tk*_ are a set of univariate time series, *k*=1,...,*K*.
- *ε*_*xt*_ is the error term.

### Li-Lee model (LL)

The Li-Lee model (Li and Lee 2005) is an extension of the Lee-Carter model to forecast multiple populations coherently.

4 $$ ln\left(m_{xti}\right) = \alpha_{xi} + \beta_{x} \kappa_{t} + b_{xi} k_{xi} + \epsilon_{xti}  $$

with

- *m*_*xti*_ is the death rates at age *x*, time *t* and population *i*.
- *α*_*xi*_ is the age-specific average of the logged death rates for population *i*.
- *β*_*x*_*κ*_*t*_ is the common factor for all populations found by applying the LC model to an average mortality of a group of population.
- *b*_*xi*_*k*_*xi*_ are the normalized first singular vectors, found by applying an SVD to the matrix *ln*(*m*_*xti*_)−*α*_*xi*_−*β*_*x*_*κ*_*t*_; they are the population-specific deviation factor from the common factor.
- *ε*_*xti*_ is the error term.

### Product-ratio model (HBY)

The product-ratio model (Hyndman et al. 2013) expand both on the LL and FDA. For a two-population application, the model is written as follow:

5 $$ f_{xti} = \text{log}\left(p_{xt} r_{xt}\right) = \mu_{x} + \eta_{x} + \sum\limits_{k=1}^{K}\beta_{tk} \phi_{xk} + \sum\limits_{l=1}^{L} \gamma_{tl} \Psi_{xl} + \epsilon_{tx}+ w_{tx}  $$

with

- *f*_*xti*_ is the smoothed logged death rates at age *x*, time *t* and population *i*, using weighted penalized regression splines.
- *p*_*xt*_ is the square root of the product of *f*_*xti*_ over population where *i*=1,2 and $p_{xt} = \sqrt {f_{xt1} f_{xt2}}$.
- *r*_*xt*_ is the square root of the ratio of *f*_*xti*_ over population, with $r_{xt} = \sqrt {f_{xt1} /f_{xt2}}$.
- *μ*_*x*_ is the age-specific mean of the product.
- *η*_*x*_ is the age-specific mean of the ratio.
- *ϕ*_*xk*_ and *Ψ*_*xl*_ are the principal components after decomposing *p*_*xt*_ and *r*_*xt*_, respectively, using the weighted principal components algorithm.
- *β*_*tk*_ and *γ*_*tl*_ are the corresponding principal component scores.
- *ε*_*xt*_ and *w*_*xt*_ are the error terms, from the product and ratio respectively.

### Multilevel functional data method (MFDM)

The multilevel functional data method (Shang 2016; Shang et al. 2016) expend on the HBY and LL model.

6 $$ f_{xti} = \mu_{x} + \eta_{xi} + \sum\limits_{k=1}^{K}\beta_{tk} \phi_{xk} + \sum\limits_{l=1}^{L} \gamma_{til} \Psi_{xil} + \epsilon_{xti}  $$

with

- *f*_*xti*_ is the smoothed logged death rates at age *x*, time *t* and population *i*, using weighted penalized regression splines.
- *μ*_*x*_ is the age-specific mean of the average mortality.
- *η*_*xi*_ is the population-specific deviation from the average mortality.
- *β*_*tk*_*ϕ*_*xk*_ is the common factor for all populations, using *K* principal component scores.
- *γ*_*til*_*Ψ*_*xil*_ is the population-specific deviation from the common trends, using *L* principal component scores.
- *ε*_*txj*_ is the error term.

The main difference between the product-ratio and the multilevel functional data methods is that the latter uses Bayesian methods to forecast and estimate the PI while the former uses the normality assumption (Shang 2016). The number of principal components are also not chosen in the same way between these two models.

### Compositional data model (CoDa)

The CoDa approach can be seen as a Lee-Carter model applied to the life table deaths (Oeppen 2008).

7 $$ clr\left(d_{xt} \ominus \alpha_{x}\right) = \beta_{x} \kappa_{t} + \epsilon_{xt}  $$

with

- *d*_*xt*_ is the life table death at age *x* and time *t*.
- *clr* is the centered log-ratio transformation, with $clr\left (d_{xti}\right) = ln\left (d_{xt}/\left [\prod _{x=0}^{X+1}d_{xt}\right ]^{1/(X+1)}\right)$.
- *α*_*x*_ is the age-specific geometric mean of the life table deaths.
- *β*_*x*_ is the first singular vector of the age mode found with an SVD applied to the matrix *clr*(*d*_*xt*_⊖*α*_*x*_).
- *κ*_*t*_ is the singular vector of the time mode multiplied by the first singular value found with an SVD applied to the matrix *clr*(*d*_*xt*_⊖*α*_*x*_).
- *ε*_*xt*_ is the error term.

### Coherent compositional data model (CoDaC)

The CoDa-coherent model expands both on the CoDa and LL models (Bergeron–Boucher et al. 2017).

8 $$ clr\left(d_{xti} \ominus \alpha_{xi} \ominus C\left[e^{\beta_{x} \kappa_{t}}\right]\right) = b_{xi} k_{xi} + \epsilon_{xti}  $$

with

- *d*_*xti*_ is the life table death at age *x*, time *t* and population *i*.
- *clr* is the centered log-ratio transformation, with $clr\left (d_{xt}\right) = ln\left (d_{xti}/\left [\prod _{x=0}^{X+1}d_{xti}\right ]^{1/(X+1)}\right)$.
- *α*_*xi*_ is the age-specific geometric mean of the life table deaths for population *i*.
- *β*_*x*_*κ*_*t*_ is the common factor for all populations found by applying the CoDa model to an average mortality of a group of population.
- *b*_*xi*_*k*_*xi*_ are the first singular vectors, found by applying an SVD to the matrix $clr\left (d_{xti} \ominus \alpha _{xi} \ominus C\left [e^{\beta _{x} \kappa _{t}}\right ]\right)$.
- *ε*_*xti*_ is the error term.

## Appendix B: Age 45 as threshold

As mentioned in the main text, we use age 45 to separate the SR peak from the hump. This age is also mentioned by Meslé (2004a) as the beginning of the hump. As an additional analysis, we also calculated a quadratic regression on *μ*_*x*_ (average SR over time) between age 25 and 60 and estimated the inflection point (or minimum) between these ages by finding the age at which the derivative of the quadratic equation is equal to 0. The average minimum among the 18 selected countries was estimated at age 45.98 with a confidence interval (CI) of 44.70–47.26.

**Fig. 11:**
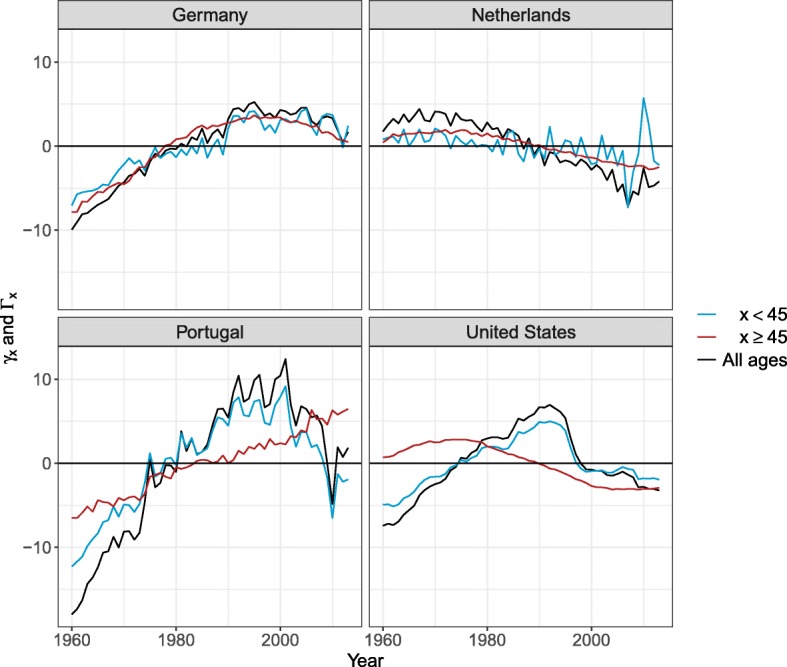
Time indices— *γ*_*t*_ in blue, *Γ*_*t*_ in red and time index for all ages in black—for Germany, the Netherlands, Portugal, and the United States

We use two age groups, because the time indices between these age groups tend to differ. Furthermore, as mentioned in the main text, a unique time index for all ages tends to be more strongly influenced by the age group 0–44, as shown in Fig. 11. However, improvements in life expectancy in recent years are mainly driven by mortality reduction at older ages (Christensen et al. 2009). Thus, separating SR trends before and after age 45 can be justified.

**Fig. 12:**
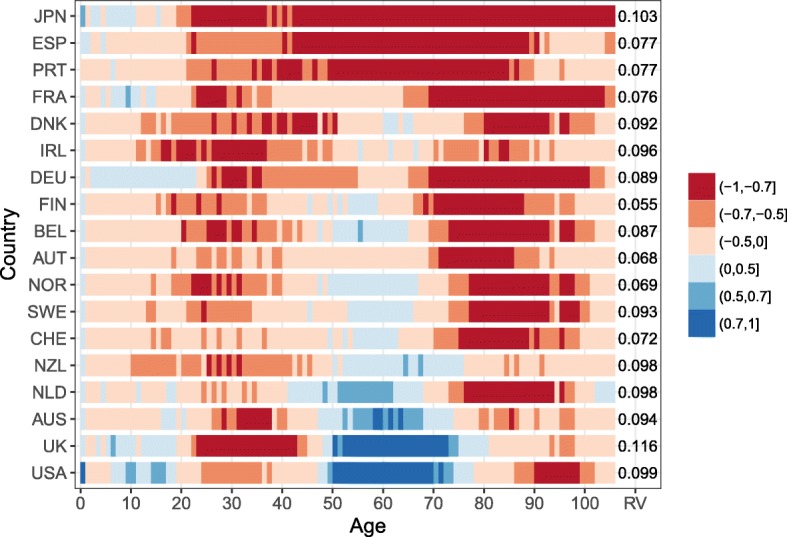
Age-specific correlation coefficients for female death rates and ratio trends over time for 18 countries and RV coefficient, 1960–2013

## Appendix C: Prediction intervals

By using the model presented in Eq. (1), two main sources of uncertainty need to be considered for the forecast: (1) errors from the SR model presented in Eq. (1), and (2) errors from the prior female forecast. For example, if we use the LC method to forecast female mortality, the female ASDR will be estimated by:

9 $$ ln\left(m_{xt}^{F}\right) = \alpha_{x} + \kappa_{t} \beta_{x} + \epsilon_{xt}^{F},  $$

where *α*_*x*_ is the average log-mortality over age; *β*_*x*_ and *κ*_*t*_ are the age profile and time index found by SVD and $\epsilon _{xt}^{F}$ is the error. The male forecast, using Eq. (1a), will then be equal to:

10 $$ ln\left(m_{xt}^{M}\right) = \alpha_{x} + \kappa_{t} \beta_{x} + \mu_{x} + I(x \leq 45)\left[\gamma_{t} \phi_{x}\right] + I(x > 45)\left[\Gamma_{t} \Phi_{x}\right] + \epsilon_{xt} + \epsilon_{xt}^{F},  $$

where *ε*_*xt*_ is the error on fitting the SR model parameters to the logged SR matrix $ln\left (\frac {m_{xt}^{M}}{m_{xt}^{F}} \right)$, as shown in Eq. (1). Equation (10) is similar to that of Hyndman et al. (2013), where the product forecast is replaced by a female forecast; only the first components are used (*K*=*L*=1) and two time indices and age profiles are estimated.

The PI are drawn based on simulations with resampled errors of the model used to forecast the time index of females (*κ*_*t*_) and of the SR (*γ*_*t*_ and *Γ*_*t*_). Assuming independence at each age between both parts of the model, the PI can be found by adding to each simulation from the female forecast, the simulations from the SR forecast, as presented in Eq. (10). The independence assumption between the two parts of the equation is reasonable, as shown below. The life expectancy is calculated for each of the simulated death rate trends and the PI are constructed using percentiles of these simulations. The uncertainty of the prior female forecast will thus be reflected in the uncertainty of the male forecast and should thus lead to wider PI for males. Many sex-independent forecast models, listed in Comparison with other models section as 1a, b, d, e, used as prior models, are also based on an SVD and time indices extrapolation, similar to the LC model. Thus, calculations based on them will follow the same principal of additive error terms in the final forecast, as in Eq. (10).

Despite the ASDR for both sexes being correlated, the trend for females and the ratio trends should be uncorrelated for Eq. (1) to be efficient. Hyndman et al. (2013) mentioned that the product and the ratio “will behave roughly independently of each other, provided that the subpopulations have approximately equal variances” (Hyndman et al. 2013, p.263). We also found that female mortality trends and the ratio trends also behave roughly independently.

Figure 12 suggests a weak or negative correlation between the females’ and ratio time trend at most ages. The negative correlation generally comes from a decrease in the females’ ASDR, but an increase in the SR. The SR time trend also tends to have a parabolic shape, leading to a weak correlation with the exponential decrease of the females’ ASDR. The RV coefficient is also weak for all countries, staying below 0.12. To assume that the ratio trends and the female trends behave roughly independently is thus reasonable.

## Appendix D: Out-of-sample evaluation

**Fig. 13:**
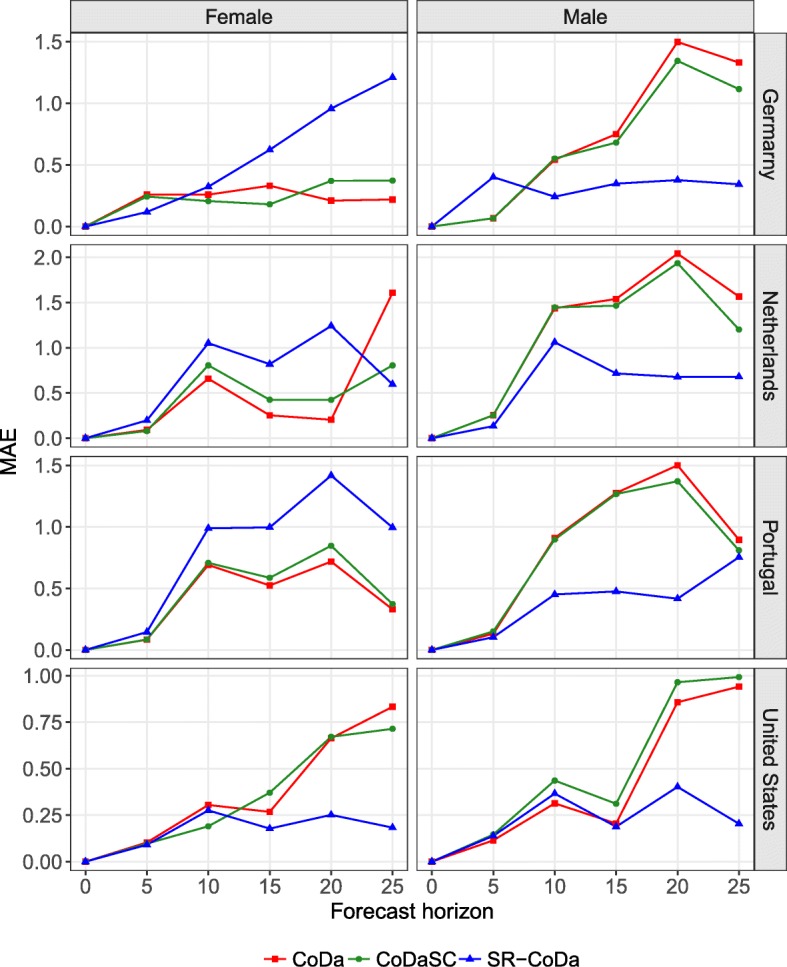
Mean absolute error (MAE) on forecasting the life expectancy at birth for a forecast horizon of 5, 10, 15, 20, and 25 years with the last year of the forecast period being 2013 with the CoDa, CoDaSC, and SR-CoDa models, for Germany, the Netherlands, Portugal, and the United States, females and males

## Appendix E: Forecasts

**Fig. 14:**
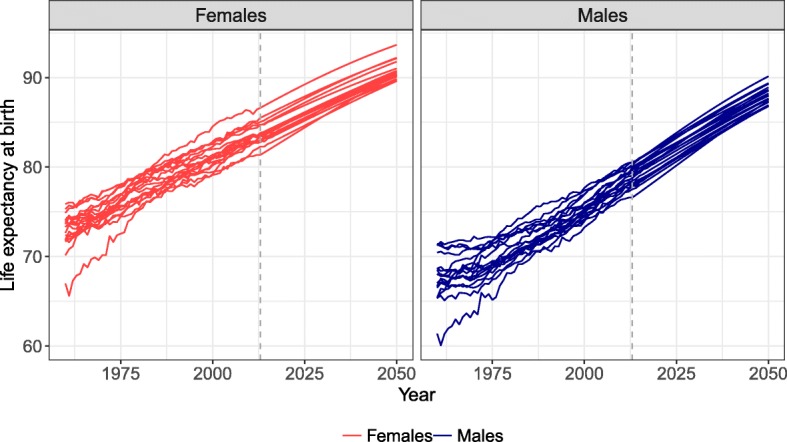
Life expectancy at birth observed from 1960 to 2013 and forecast from 2014 to 2050 with the CoDaCC model for females and with the SR-CoDaCC model for males, 18 countries

## Abbreviations

ASDR: Age-specific death rates
CoDa: Compositional data analysis forecast model
CoDaCC: Compositional data analysis forecast model extension for country-coherent forecast
CoDaSC: Compositional data analysis forecast model extension for sex-coherent forecast
LC: Lee-Carter
LCCC: Lee-Carter model extension for country-coherent forecast (Li and Lee 2005)
LCSC: Lee-Carter model extension for sex-coherent forecast (Li and Lee 2005)
LL: Li-Lee model, or Lee-Carter model extension for multiple populations forecasts, with unspecified populations
HBY: Hyndman, Booth and Yasmeen product-ratio model
MAE: Mean absolute error
ME: Mean error
MFDM: Multilevel functional data method (Shang 2016)
OSC: Other sex-coherent models (includes the LCSC, CoDaSC, HBY, and MFDM models)
PI: Prediction intervals
R: Pearson’s correlation coefficient
SR: Sex ratio
SR-FDA: Sex ratio model based on the FDA model as prior
SR-LC: Sex ratio model based on the LC model as prior
SR-LCCC: Sex ratio model based on the LCCC model as prior
SR-CoDa: Sex ratio model based on the CoDa model as prior
SR-CoDaCC: Sex ratio model based on the CoDaCC model as prior
SVD: Singular value decomposition
UN: Probabilistic forecast method used by the United Nations Raftery et al. 2012, 2014

## Footnotes

1 The HMD provides data for Germany starting in 1990 only, but offers data for East and West Germany separately since 1956. To have a longer time series for Germany, we combined death counts and exposure to risk data for East and West Germany.
